# Prospective effects of food safety trust on brand evangelism: a moderated-mediation role of consumer perceived ethicality and brand passion

**DOI:** 10.1186/s12889-023-17268-1

**Published:** 2023-11-24

**Authors:** Zhou Guanqi, Zeb Un Nisa

**Affiliations:** 1 School of Economics, Yunnan University, Kunming, China; 2 Institute of Business & Management, University of Engineering and Technology, Lahore, Pakistan

**Keywords:** Food safety Trust, Brand passion, Consumer perceived ethicality (CPE), Brand evangelism, Ready to eat food, Sternberg theory of love

## Abstract

**Background:**

The current study integrates brand management literature with food consumption research and develops an integrative framework by combining food safety trust, consumer perceived ethicality, brand evangelism, and brand passion into a single conceptual model.

**Method:**

This quantitative study included 228 ready-to-eat consumers in China using purposive sampling. Data were collected at two periods in time, resulting in a time-lag study in which respondents provided data on independent and moderating variables at time 1 and mediating and dependent variables at time 2 with the same respondents from time 1. The hypothesized correlations were tested using SEM and PROCESS Macro techniques.

**Results:**

According to the findings, trust in food safety has a significant impact on brand evangelism and passion. Furthermore, consumer perceived ethicality (CPE) found to have a substantial moderating role between food safety-FS and brand passion. Moreover, we validated the brand passion role as a mediator between brand evangelism and food safety trust, and investigated whether consumer perceived ethicality conditionally affects the strength of the indirect relationship among food safety trust and brand evangelism through brand passion, indicating a moderated-mediation mechanism.

**Originality:**

Drawing on Sternberg theory of love, current study is the first of its kind to evaluate the boundary role as well as the conditional indirect influence of customer perceived ethicality among the study’s variables and provides useful information for ready-to-eat food brand managers on how to keep them interested in their risk-free food products.

## Background

In the past two decades, consumers’ nutritional preferences have undergone a paradigm shift, and several studies on the growing interest in the ready-to-eat food sector have been carried out in both developed and developing nations [1]. Consumers today frequently display their passion for a brand of food product they purchase. A recent large-scale survey of 16,000 consumers across eight countries found that 81% of participants had to rely on brands when making decisions, but that just 34% of consumers felt faith in the bulk of the goods they buy or use [2]. Within the food sector, trusted brands are critical in influencing consumers to buy food goods because they give key indications about food characteristics (such as quality and safety) that consumers cannot directly view [3–5]. Promoting consumer passion in food brands is especially important now that food safety concerns have eroded consumer trust in food [6, 7]. Sustaining desirable food safety is the way to be succeeded in the business because it is one of the most important things that customers consider when choosing their food. To ensure food safety, an increasing number of individuals are focusing on the quality of their food.

China’s large and densely populated population has led to significant public attention on food safety issues. China is the second-largest food importer in 2019, with a total value of US$ 11.66 billion, representing a 34.3% increase compared to the previous year [8]. In 2018, the highest market share (22%) of China’s imported food items came from imported fruit [9]. Trust in food safety has a significant impact on Chinese consumers’ preferences for food brands [10, 11]. As they believe that such foods have better safety trust and provide consumers with supreme meaning, Chinese consumers typically show more desire and enthusiasm for food brands with good safety than low safety [12]. However, the underlying mechanism of the influence of food safety is unclear, especially from the standpoint of brand managers. For instance, satisfied customers are frequently seen as diversified, and they are not only excited about the brand but also serve as a source of good recommendations [13]. It is crucial to examine how Chinese customers’ brand loyalty and evangelism in the ready-to-eat food category are shaped by food safety.

The current work intends to bridge this gap by creating an integrative framework that uses food safety to forecast consumer brand passion, which in turn forecasts consumer brand evangelism for ready-to-eat food. We specifically use the Sternberg duplex theory of love, which states that love has three distinct elements: closeness, choice/commitment, and desire. “The drives that lead to romance, physical attraction, sexual completion, and associated phenomena in loving relationships,“ according to Sternberg, are all examples of passion [14, 15]. Additionally, despite the recent expansion of literature in this research area, [16, 17], it is essential to highlight the existing gap in research concerning the impact of food safety trust on positive outcomes i.e., brand passion and evangelism. This study seeks to contribute to the field of consumer psychology research by empirically examining how brand passion and evangelism are subsequently impacted by food safety trust and whether customer perceived ethicality may initiate such a relationship between food safety trust, brand passion, and brand evangelism in a moderated-mediation model. The current study contributes to the domains of branding, food safety, and consumer psychology, especially by discussing two essential concepts: customer brand passion and brand evangelism. To the best of our knowledge, this is the first study to investigate the effect of food safety trust on brand passion and evangelism in a ready-to-eat food scenario. It identifies how food safety trust boosts brand passion, which in turn enhances brand evangelism.

## Literature review and hypothesis development

Sternberg’s triangle theory was employed by the researchers to explain why individuals become passionate regarding specific foods. Initial qualitative research compared intense feelings for a brand or product to extreme feelings for another person, such as romantic relationships among individuals [18]. According to a recent definition, brand passion is “a psychological construct comprised of excitation, attraction, and obsession for a brand” and “a emotion that few customers embrace” [19]. Prestige for brand, brand distinctiveness, self-expressive and hedonic aspects is few of the antecedents and outcomes associated with brand passion that have been studied. In similar vein, brand identity and brand trust, is the strongest indicator of brand love [20]. Prior studies have demonstrated that brand loyalty results from consumer love for a product or service [21]. Furthermore, contradictory results have called into question whether a consumer’s willingness to pay a premium for a brand is influenced by their level of brand passion ^19^.

### Relationship between food safety, brand passion and brand evangelism

In literature, brand evangelists and food safety have a significant association [22]. According to Matzler, Pichler and Hemetsberger 23, brand evangelism refers to actively and passionately promoting a brand and persuading others to engage with it (p. 27). In line with Sternberg’s triangular theory, consumers develop relationships with consuming objects (i.e., goods, stores, brands, etc.) that might vary from feelings of hostility to a tiny bit of fondness to what would be considered love between people. Brand evangelism is seen as an advanced kind of word-of-mouth communication. Prior research has established a correlation between food safety trust and service outcomes, such as word-of-mouth promotion [24] and intention to purchase [24]. Similarly, researchers studied the link between brand trust and brand evangelism and revealed that trust encourages evangelistic activity [24–26]. Consumers can utilize media to share their shopping experiences with food goods through written texts, images, videos, or even applications. Visually enhanced material makes food safety information more entertaining and appealing, promoting the rapid spread of food safety data to a vast population [27, 28]. Furthermore, the advice of opinion leaders and peers has substantially aided in the dissemination of food safety information and effectively boosted consumer contact, significantly alleviating the issue of misinformation between consumers and food suppliers. According to Al-Harbi and Badawi [29], opinion leaders enable the instantaneous distribution of food safety information, boosting consumers’ knowledge of risks and facilitating swift solutions to emergent risk issues. This assists in identifying potential food safety hazards and effectively responding to public misconceptions in specific scenarios. These studies show the critical function of referrals (evangelists) in transmitting food safety knowledge and increasing global food safety. Based on aforementioned literature, it is thus proposed:

#### H1

Food safety trust has significant positive effect on brand evangelism dimensions i.e., (a) brand purchase intention, (b) positive brand referrals, and (c) oppositional brand referrals.

Within the realm of marketing scholarship, the origin and outcomes of brand passion have been elucidated, aiding in comprehending consumer behavior [30]. Brand passion, as corroborated by Bauer, Heinrich and Martin [31], denotes an emotional bond to a brand. Notably, the consumer experience is a potent catalyst for evoking emotions that can in turn elevate consumer engagement [32]. According to Pourazad, Stocchi and Pare [33], brand passion is significantly related to brand advocacy. However, it’s noteworthy that passion for a brand, though intense, shouldn’t overshadow other facets of identity.

The nexus between brand passion and word-of-mouth (WOM) communication is evident in research, elucidating its role in fueling evangelism [23, 34–36]. Brand-loyal patrons might mistakenly perceive a mutual affection with the brand [37]. Capitalizing on social media interactions, a company can amplify consumer engagement, reinforcing beliefs in reciprocity and catalyzing evangelical behaviors [34]. Thus, a passionately engaged consumer not only forges a deeper emotional bond with the brand but also experiences a sense of void in its absence.

Belk, Ger and Askegaard [38] expound on how conventional consumption is intrinsically motivated by desire, outlining a nexus between passion and specific behaviors. This conceptualization aligns with the notion of brand evangelism, wherein consumers ardently advocate for a brand’s cherished attributes and positive associations, aimed at those yet to appreciate its allure. Brand evangelists, driven by devotion, feel compelled to share their fervor with others. This inclination mirrors the notion of strong brand passion, wherein consumers gravitate towards brands they feel a profound sense of comfort and affinity for [39, 40]. Therefore based on above discussion following hypothesis is formed.

#### H2

Brand Passion has significant positive effect on brand evangelism dimensions i.e., (a) brand purchase intention, (b) positive brand referrals, and (c) oppositional brand referrals.

Food quality encompasses the amalgamation of attributes that provide satisfaction to customers and influence various stakeholders, encompassing anticipated and unforeseen effects, as defined by the International Organization for Standardization’s food quality indexes [41]. Scholars spanning diverse disciplines have delved into factors pivotal for food safety, including brand love and brand loyalty [42]. Characterized by a profound connection to a particular brand, brand passion is associated with its prestige, evoking happiness and enthusiasm [42, 43]. Prior investigations have shown that brand love correlates with heightened sales, as emotionally attached consumers tend to make more purchases [44, 45].

In recent years, global incidents concerning food safety have gained significant attention. According to the World Health Organization (WHO), there were over 600 million instances of food-related illnesses and 420,000 fatalities due to food poisoning in 2010 [40]. As established by Cuesta-Valino, Gutierrez-Rodriguez, Sierra-Fernandez and Aguirre Garcia [46], food safety distinctly influences spending willingness. Furthermore, Yoo, Lee and Jeon [47] established a strong link between positive attitudes and trust in food safety. Prior research underscores the significance of healthiness and taste in shaping consumer attitudes; however, these factors have received limited scrutiny concerning ready-to-eat food products and their nexus with brand passion. Hence, the following hypotheses was proposed:

#### H3

Food safety trust has significant positive effect on Brand Passion.

### Mediating role of brand passion

According to the Sternberg theory of love, customers’ emotions are influenced by intimacy and passion, that might drives their behavior. In line with this argument, favourable emotions, such as attraction and passion, mediate the favorable effect of food product qualities on positive consumer behavioral outcomes [48]; quality of product, atmospherics, and sales personal behavior on buying intention [49, 50]. In the fashion apparel sector, atmospherics and hedonic pleasures influence customers’ favorable feelings such as passion, which in turn influences brand advocacy [51]. Similarly, the influence of food quality on purchasing intent and WOM is mediated by customer emotions [52]. In similar vein, it becomes a reasonable deduction that passionately engaged consumers are inclined to evolve into brand evangelists, propelled by the compelling attributes of food quality, leading to a range of favorable outcomes. Therefore, it is expected that trust in food safety will influence brand passion, that will promote brand evangelism. As a result, the following hypotheses was proposed:

#### H4

Brand Passion mediates the relationship between food safety trust and brand evangelism dimensions i.e. (a) brand purchase intention, (b) positive brand referrals, and (c) oppositional brand referrals.

### The role of consumer perceived ethicality

The intersection of brand management and consumer ethics is exemplified in the realm of ethical branding. In the context of multiple stakeholders, an ethical brand is one that conducts itself with responsibility, integrity, respect, and accountability [53]. Scholars consistently advocate that companies should aspire to establish an ethical identity, aligning with consumer expectations in an era where ethical considerations hold increasing sway [54]. This trend is underscored by the contemporary environment, where consumers increasingly favor brands that address ethical concerns [55].

Building upon this backdrop, recent research has introduced the concept of “customer perceived ethicality” as a significant dimension [56]. This concept encapsulates consumers’ overarching perception of the moral integrity of entities such as businesses, brands, products, or services [57]. In essence, it encapsulates the extent to which consumers view a particular product or brand through an ethical lens. Aligning with theories of emotional connection, the consumer-brand relationship is akin to a bond, characterized by emotional resonance [58]. In the context of the present study, it can be proposed that consumers who harbor robust ethical perceptions of a brand’s safety commitments may foster stronger brand passion compared to those with lower ethical perceptual levels. Therefore, we hypothesize that:

#### H5

Consumer perceived ethicality plays a boundary role in the relation between brand passion and food safety so that the relation is stronger for people who have higher levels of consumer perceived ethicality (CPE) compared to those who have low.

### Conditional indirect effect

**Fig. 1:**
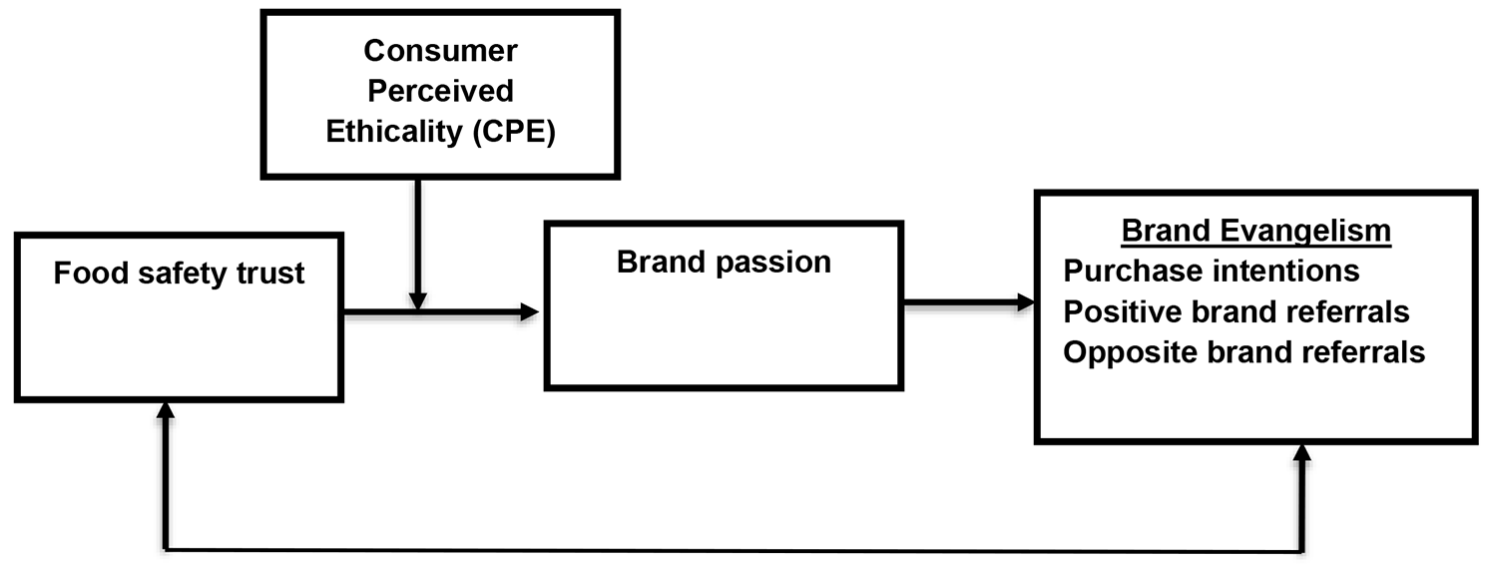
Research Model

Because we postulate that consumer perceived ethicality-CPE plays a boundary role between food safety (IV) and brand passion (Mediator), we hypothesize that consumer perceived ethicality-CPE may have a conditional impact on the strength of the indirect relationship between food safety and brand evangelism via brand passion suggesting a moderated mediation pattern among the variables. Figure 1 depicts food safety as an independent variable that has a positive impact on brand evangelism via brand passion, while consumer perceived ethicality plays a boundory role in the relationship between (IV) and (DV).

#### H6

Consumer perceived ethicality plays a boundary role in the relataionship between food safety and brand evangelism via brand passion in such a way that the relatiosnhip will be stronger in case of high consumer perceived ethicality. This hypothesis should be placed before Fig.1

## Materials and methods

We employed a purposive sampling approach, targeting respondents from the three largest metropolitan cities in South China. This approach aimed to include individuals who are more reliant on ready-to-eat food (university students, policemen, and construction workers). By deliberately selecting participants from these urban areas known for their increased consumption of such foods, we sought to reduce the potential for sampling bias and enhance the representativeness of our study’s findings. The data collection process involved a two-part questionnaire survey. The initial phase focused on variables related to food safety and its outcomes, particularly examining the influence of brand passion on outcomes. Data collection occurred in two distinct stages: time 1 encompassed the assessment of food safety and consumer perceived ethicality, while time 2 encompassed the evaluation of brand passion and brand evangelism, thus creating a time-lagged study design. In the subsequent phase, essential demographic information such as gender, age, occupation, education, and income were gathered. The distribution of survey questionnaires was strategically aimed at university canteens, public places frequented by policemen, and construction sites. These respondents were chosen based on their adherence to a tight schedule and likelihood to consume ready-to-eat packaged food. Respondents’ inputs were based on their genuine thoughts during the purchasing process.

The survey conducted between May and June 2022 yielded a total of 350 responses, which included 23 participants with limited ready-to-eat food consumption experiences at work. Rigorous data integrity checks were performed, leading to the exclusion of 5 responses due to inadequate data quality. Consequently, a usable response rate of 65% was achieved. The research finally focused on a sample size of 228 Chinese respondents. Demographically, 71.9% of the participants were male, with 52.6% falling within the age range of 20 to 30. Furthermore, 58.8% were married, 46.1% possessed a master’s degree, and 36.8% reported an income exceeding 51,000 RMB (1 RMB = 0.14 US dollar).

The scales of each variable were constructed by reviewing the existing literature and depending on the fairly mature scales to determine that the five variables that were studied can be assessed accurately. Furthermore, in order to better meet the features of this study, the variable items were adjusted based on the study’s content. A 7-point Likert scale was used to evaluate the measures in this study. A ten-item scale was used to assess food safety trust adopted from Medeiros, Hillers, Chen, Bergmann, Kendall and Schroeder [59], “I am worried that I may get sick if I eat hot dogs right out of the package.” Brand passion is measured using a five-item scale adopted from Thomson, MacInnis and Park [60] “I am passionate about this brand.“ We used 10 items to measure the brand evangelism dimensions: four for BPI, three for PBR, and three for OBR, all derived from Becerra and Badrinarayanan [61]. Consumer perceived ethicality [62] (CPE) was measured using six items scales and sample questions are “The brand is a socially responsible brand, The brand seems to make an effort to create new jobs”.

## Results

### Common method bias test and confirmatory factor analysis (CFA)

We employed two techniques to address common method bias (CMB): Harman’s one-factor test and the marker variable method. The application of Harman’s one-factor test indicated that a single factor could account for only 25% of the variance in the sample, implying the absence of significant CMB. Furthermore, we introduced a common latent factor (CLF) associated with all items to detect any potential method bias, and the results reinforced the absence of CMB.

In AMOS 22.0, we used CFA to test the discriminant validity of the food safety, consumer perceived ethicality (CPE), brand passion, and brand evangelism variables. All objects were exposed to CFA in the first stage [63]. The results showed a good fit to the data (χ^2^[df] = 028.75[319]; RMSEA = 0.06; NFI = 0.79; TLI = 0.93; CFI = 0.78). Loadings ranging from 0.38 to 0.88 were significant, as shown in Table 1. The average variance extracted (AVE) by FST, BP, BPI, PBR, and OBR, respectively, was 0.50, 0.60, 0.69, 0.65, and 0.61. The above-mentioned fit statistics, substantial loadings, and average variance recovered by each latent construct (> 0.50) all confirmed convergent validity [64]. The discriminant validity criteria proposed by [65] was also tested. In general, the AVE of each latent variable was greater than the equivalent correlation between the variables in Table 2. The composite reliability ratings were more than 0.70, indicating that all measurements were trustworthy [66]. The summarized data and correlations were provided in Table 3.

**Table 1:** Results of confirmatory factor analysis

Latent construct	Items	Factor load	CR		AVE
**Food safety trust** *α = 0.831*	Counter-thawing for ready-to-eat food is safe.	0.68	0.91		0.51
	Firm egg yolks and whites’ matter for safety.	0.69			
	Pasteurized apple juice is a safety priority.	0.66			
	Cleanliness after raw meat is vital for ready-to-eat food.	0.73			
	No concern about meat thermometer use.	0.67			
	40°F fridge doesn’t worry me for ready-to-eat safety.	0.79			
	Raw sprouts safety isn’t a concern.	0.80			
	I am worried that I may get sick if I eat hot dogs right out of the package.	0.66			
	Pasteurized dairy is my safety choice.	0.74			
	Raw oyster illness concern for me.	0.71			
**Brand passion** *α = 0.825*	I feel a strong passion for this brand.	0.87	0.88		0.602
	I have genuine confidence in this brand.	0.82			
	I share a deep connection with this brand.	0.83			
	This brand strongly appeals to me.	0.80			
	This brand brings me immense delight.	0.50			
**Brand evangelism** *Brand purchase intentions* *α = 0.792*	In future, I am inclined to purchase the brand.	0.66	0.86		0.624
	In future, I plan to acquire brand X.	0.70			
	In future, it’s probable that I’ll make a purchase from the brand.	0.98			
	In future, I might make a purchase from the brand.	0.78			
*Positive brand referrals* *α = 0.775*	I share positive recommendations about the brand.	0.81			
	I suggest the brand to my friends.	0.82	0.84		0.645
	If my friends were searching for X, I would advise them to choose the brand.	0.78			
*Oppositional Brand Referrals (OBR)* *α = 0.787*	If my friends are seeking food, I would advise against purchasing any other brands.	0.71	0.83		0.622
	I might share unfavorable word of mouth about the other brands.	0.81			
	I will not recommend brand to my peers	0.84			
**Consumer perceived ethicality**	The brand upholds ethical principles.	0.74	0.857	0.50	
*α = 0.793*	The brand consistently follows legal regulations.	0.71			
	The brand demonstrates social responsibility.	0.69			
	The brand refrains from harmful actions under all circumstances.	0.67			
	The brand is reputable.	0.73			
	The brand evaluates outcomes thoroughly before making decisions, considering impacts on all stakeholders.	0.70			
	**Fit indices**				
	χ ^2^[df] = 028.75[319]; RMSEA = 0.06; NFI = 0.79; TLI = 0.93; CFI = 0.78				

**Table 2:** Discriminant Validity

constructs	1	2	3	4	5	6
Food safety trust	0.699					
Brand passion	0.613	0.777				
Brand purchase intentions	0.580	0.750	0.832			
Positive brand referrals	0.506	0.460	0.487	0.807		
Opposite brand referrals	0.541	0.698	0.674	0.787	0.778	
Perceived ethicality	0.432	0.385	0.294	0.391	0.581	0.49

**Notes**: The extracted average variance was depicted on the diagonal

**Table 3:** Means, Standard Deviations with Correlations

Variables	Mean	Std.D	1	2	3	4	5	6
1. Food safety trust	4.78	1.18	1					
2. Brand passion	4.21	1.56	0.32^**^	**1**				
3. Brand purchase intentions	4.47	1.61	0.64^**^	0.56^**^	1			
4.Positive brand referrals	4.41	1.46	0.47^**^	0.60^**^	0.48^**^	1		
5.Oppositional brand referrals	4.92	1.34	0.52^**^	0.38^**^	0.70^**^	0.33^**^	1	
6.Perceived ethicality	4.17	1.18^*^	0.34^**^	0.51^**^	0.46^**^	0.31^*^	0.27^**^	1

N = 228; *p < 0.05; **p < 0.01

### Hypotheses testing

We utilized SPSS 22 software along with the PROCESS macro to rigorously test the study hypotheses. Furthermore, we employed the four-step procedure introduced by [67]. In the first phase of this procedure, we examined the links between Food Safety Trust (FST) and brand evangelism. The results demonstrated a significant positive association between FST and Brand Purchase Intentions (BPI) (B = 0.70, *p* < 0.05), Positive Brand Referrals (PBR) (B = 0.39, *p* < 0.05), and Opposite Brand Referrals (OBR) (B = 0.51, *p* < 0.05), thus supporting hypotheses H1a, H1b, and H1c. Moving on, the second phase involved evaluating the relationship between FST and brand passion, where a statistically significant connection was observed (B = 0.43, *p* < 0.05), confirming hypothesis H2.

Furthermore, during the third phase, we assessed the links between brand passion and brand evangelism. The results indicated a positive correlation between brand passion and BPI (B = 0.40, *p* < 0.05), PBR (B = 0.47, *p* < 0.05), and OBR (B = 0.20, *p* < 0.05), thereby validating hypotheses H3a, H3b, and H3c. However, it’s noteworthy that introducing brand passion as a mediator in the relationships between FST and brand evangelism yielded substantial associations in the results. These findings, detailed in Table 4, showed that brand passion significantly mediated the relationships, as evidenced by the associations with BPI (B = 0.17, *p* < 0.05), PBR (B = 0.20, *p* < 0.05), and OBR (B = 0.09, *p* < 0.05), providing additional support for hypotheses H4a, H4b, and H4c.

**Table 4:** Mediation analysis

		Brand passion			Brand purchase intentions			Positive brand referrals			Opposite brand referrals		
		B	SE	R^2^	B	SE	R^2^	B	SE	R^2^	B	SE	R^2^
1	Food safety trust	0.43**	0.08	0.11	0.70***	0.06	0.55	0.39**	0.06	0.45	0.51**	0.06	0.32
2	Brand passion	--	--	--	0.40**	0.04	0.55	0.47**	0.04	0.45	0.20**	0.05	0.32
IE (bootstrap)					0.17** [0.08 0.29]			0.20** [0.11 0.31]			0.09** [0.04 0.17]		
IE (normal distribution)					0.17** (4.36)			0.20** (4.53)			0.09** (3.15)		

Note: N = 228. 95% (CIs) 5,000 bootstrap samples. Statistically significant IE (indirect effects) are in bold face text. *p < 0.05, **p < 0.01, ***p < 0.001

Hypothesis 5 postulated that the association between Food Safety Trust and brand passion would be contingent on the level of consumer perceived ethicality. As revealed, the significant relationship between Food Safety Trust and Perceived Ethicality exhibited notable implications for Brand Passion (B = 0.13, SE = 0.04, *p* < 0.001). Employing Bootstrap analysis, the conditional direct effects of the independent variable (IV) on the dependent variable (DV) were established across varying degrees of the moderator, particularly in the context of high Consumer Perceived Ethicality, specifically for Brand Passion (as illustrated in Table 5). The interaction plot further substantiated the hypothesized moderation effect of Consumer Perceived Ethicality on this relationship.

**Table 5:** Moderated Regression Analysis Results

Predictors	Brand Passion				
	R	R^2^	Estimate	SE	
Step-1	0.62***	0.38***			
Constant			3.16***	0.57	
FST			0.02	0.15	
CPE			0.44**	0.15	
Step 2	**∆R²**	0.04*			
FST x CPE			0.13***	0.04	
**Conditional direct effects of X on Y at values of Moderator (i.e. Consumer Perceived Ethicality, CPE)**					
**FNE**	**Effect**	**Boot SE**	**LLCI**	**ULCI**	
CPE high	0.65***	0.07	0.51	0.80	
CPE Moderate	0.49***	0.05	0.40	0.59	
CPE low	0.34***	0.07	0.21	0.47	

**Note**: n = 228. Unstandardized regression coefficients are reported. Bootstrap sample size = 5000; LL = lower limit; CI = confidence interval; UL = upper limit. Where FST = Food safety trust, CPE = Consumer Perceived Ethicality, PB = Brand Passion. *p < 0.05, **p < 0.01, ***p < 0.001

Importantly, the nature of the interaction between Food Safety Trust and Brand Passion demonstrated variability across different levels of Consumer Perceived Ethicality (Low, Moderate, and High). Consistent with the prediction, when Consumer Perceived Ethicality was low, the positive relationship between Food Safety Trust and Brand Passion was attenuated (B = 0.34, t = 5.16, *p* < 0.001), and notably, even displayed a negative association. Conversely, this relationship was more robust and positive when Consumer Perceived Ethicality was high (B = 0.65, t = 9.16, *p* < 0.001). Thus, the empirical findings provided support for Hypothesis 5, indicating that Consumer Perceived Ethicality indeed moderates the impact of Food Safety Trust on Brand Passion.

## Discussion

One of the most significant worldwide health challenges today is food safety. Most people also take food-related chemical compounds, preservatives, and additives into account when thinking about food safety. “Food shall be non-toxic, harmless, comply to nutritional needs, and shall not cause any acute, subacute, or chronic harm to human health” in the most recent amendment of the “Food Safety Law of the People’s Republic of China” [68]. Passionate customers spread the word. Passion, on the other hand, is not something that is inherently bad [69], but instead the product of several other factors, one of which being the ethical concerns of the consumer. This assertion is supported by our data, which also has important implication for marketing and theory. When ethical shoppers are passionate brand advocates, we can agree that they are the most crucial and effective brand advocates. Although loyalty can take many forms, brand advocacy has the added benefit of credibility and the ability to develop powerful brand communities. However, according to Zhang, Husnain, Usman, Akhtar, Ali, Khan, Abbas, Ismail, Rehman and Akram [70] enthusiastic consumers may also be the most committed opponents when the brand disappoints them. As a result, as Fournier [71] suggests, marketers must actively engage connections with passionate customers and participate in adequate and authentic interactions on a frequent basis. Another notable observation is that ethical customers appear to engage in more evangelizing than others. This research demonstrates that such people are actually communicative. As a result, if ethical consumer is no more enthusiastic, she/he will engage in word-of-mouth transmission. As a result, we can argue that ethical consumers are crucial brand advocates.

Earlier research in China focused on food safety events, hazardous brand, safety in supply chain, country of origin as a quality parameter, and sustainable aspects such as ethical, social, and environmental concerns [11, 72–74]. Other literature reveals on consumer purchasing decisions about health, the environment, and food safety, as well as safety incidence, particular food product safety [16, 75]. Present research reveals that food safety trust positively related to brand evangelism. The past studies reported that brand engagement works as underline mechanism between value co-creation and brand evangelism [76]. Thus the results are aligned with past studies [76, 77]. According to the findings, brand evangelism and food safety trust are related through brand passion. There hasn’t been much research on this subject, regardless of the fact that consumers passion has been identified as the essential aspect of brand love that matters the most for marketing managers. In addition, marketing professionals heavily influenced the brand passion construct by Sternberg’s theory on interpersonal love. As a result, consumers’ affection for a brand has been compared to the emotion felt between two people. Prior definitions of passion indicated that it was rare and of an obsessive character. However, we contend that the popularity and consumer passion around companies like Google, Microsoft, and Tiktok, among others, show that a customer’s enthusiasm for a preferred brand is distinct from a romantic devotion. Brand passion is significantly more prevalent than past academic study revealed, and it doesn’t necessarily seem to be obsessive. The results are aligned with past studies which reported that brand passion mediates the links [78]. Finally, in two ways, our research adds to the growing body of knowledge about brand evangelism [79]. Our findings firstly validate that evangelism is a different form of brand advocacy that requires a separate line of investigation by showing that brand passion and evangelism have distinctive relationship quality bases. Second, our study’s result that food safety trust affected evangelism adds to the growing evidence that it is a deep functional feature built on trust that necessitates the development of emotional relationship quality.

## Implications of the study

Marketers must acknowledge that consumer passion for products or services evolves gradually through experiences. Customers have a sense of ownership and a right to voice their support for brands. Therefore, it is crucial for marketers to actively foster brand advocacy by equipping customers with the necessary tools to connect with and develop affection for the brand. The findings of this study offer valuable insights to managers for effective brand management, particularly in the context of food safety and ready-to-eat products.

Food safety trust assumes an elevated significance, not only in terms of enhancing the frequency of purchase but also in strengthening brand advocacy. In the realm of ready-to-eat products, ensuring food safety becomes a paramount concern. Brand evangelists, driven by their genuine passion, play a pivotal role in advocating for products with reliable safety standards. Their endorsement and positive word-of-mouth communication serve as powerful tools to influence potential customers, especially in a sensitive domain like food safety.

Creating online platforms, particularly on social media, facilitates direct customer-brand interaction. This engagement avenue becomes especially relevant in the context of ready-to-eat food products, where customers seek reassurance and real-time responses to queries about safety measures. By fostering brand passion and commitment, marketers can effectively address consumer concerns, leading to increased trust and loyalty. Furthermore, as this research highlights, customers’ ethical perception of a brand significantly correlates with their level of passion. In the case of ready-to-eat food, ethical considerations play a substantial role as customers demand transparency and integrity in terms of food sourcing, processing, and safety measures. Thus, brand managers should align marketing strategies with ethical values to resonate with customers and intensify brand passion.

## Limitations and future research directions

Numerous constraints should be considered when interpreting research findings. This is a cross-sectional study, however the relationship between customers and brands can vary over time, such as when a customer begins to love a brand or leaves the brand. Longitudinal research methods can be used to explore relationships throughout time. To analyze customer participation in ready-to-eat foods, we used a standardized questionnaire. Given the possibility of limiting participant expression, future work may use a mixed-methods approach. As present study was conductd in developing context of China, in order to further improve generalizatibilty and representativeness of present study’s findings, future studies can replicate our model with enhanced sample size from similar cross cultual settings. Further, this study was limited to the hotel business. As a result, future research might include additional sectors to study the proposed approach. Finally, the scope of this research is confined to investigating the function of brand passion in boosting brand evangelism as well as the influence of its dimensions. As a result, future research might include cognitive processes (such as brand trust, customer happiness, and so on) with emotional mechanisms to offer in depth understanding of the involvement of cognitive and emotional qualities in the FST-brand evangelism relationship.

## Conclusions

Based on the Sternberg theory of love, present research uses SEM and PROCESS Macro techniques to analyze the influence of food safety trust on brand evangelism, and it validates through eamining the mediating role of brand passion and the moderation of consumer perceived ethicality. Food safety was found to have a substantial impact on evangelism, that is mediated by brand passion. Furthermore, consumer perceived ethicality functions as boundary condition among FST and brand evangelism. The higher and more positive the effects of FST on brand evangelism, the better the consumer perceived ethicality. Present research also adds to literature by analyzing consumer perceptions of ethicality and how they conditionally influence the intensity of the indirect effect among food safety trust and brand evangelism through brand passion, implying a moderated mediation configuration between variables in the study. The current study proposes ready-to-eat food product marketing approaches as a consumer-brand relationship principle. Producers and marketers of ready-to-eat food products are given advice on how to create effective programs to attract customers and facilitates the long-term success of their products.

## Data Availability

The datasets used and/or analyzed during the current study are available from the corresponding author on reasonable request.
